# Evaluation of antibiotics in pediatrics using the defined daily doses method and the World Health Organization (WHO) access, watch, and reserve classification (AWaRe 2021): a cross-sectional study

**DOI:** 10.11604/pamj.2024.48.88.36498

**Published:** 2024-07-04

**Authors:** Heni Lutfiyati, Jarir At Thobari, Nanang Munif Yasin, Zullies Ikawati

**Affiliations:** 1 Faculty of Pharmacy, Doctoral Program in Pharmaceutical Sciences, Universitas Gadjah Mada, Yogyakarta, Indonesia,; 2 Department of Pharmacy, Faculty of Health Science, Universitas Muhammadiyah Magelang, Magelang, Indonesia,; 3 Department of Pharmacology and Therapy, Faculty of Medicine, Public Health and Nursing, Universitas Gadjah Mada, Yogyakarta, Indonesia,; 4 Center for Child Health, Faculty of Medicine, Public Health and Nursing, Universitas Gadjah Mada, Yogyakarta, Indonesia,; 5 Department of Pharmacology and Clinical Pharmacy, Faculty of Pharmacy, Universitas Gadjah Mada, Yogyakarta, Indonesia

**Keywords:** Antibiotic, pediatric, automatic stop order

## Abstract

**Introduction:**

irrational antibiotic use can result in antibiotic resistance, which, in turn, can lead to increased morbidity and mortality, as well as high treatment costs. This phenomenon is more common in children because they are a population that often receives antibiotics. This study aimed to evaluate antibiotic use in pediatric patients in Indonesia using the Defined Daily Doses (DDD) method and the WHO Access, Watch, and Reserve Classification (AWaRe 2021).

**Methods:**

this is an observational study that uses a quantitative approach to calculate the quantity of antibiotic use in pediatric patients in two hospitals in Central Java, Indonesia. A cross-sectional study was conducted at two referral hospitals in Central Java Province, Indonesia, from January to December 2020. The DDD approach was used to examine antibiotic use. Antibiotic use was also classified into three groups based on the World Health Organization´s “AWaRe” categorization: “Access,” “Watch,” and “Reserve.”

**Results:**

a total of 505 pediatric encounters were assessed. The most frequently prescribed antibiotics in pediatric inpatients were cefotaxime accounting for 42.72%, ceftriaxone 22.91% and ampicillin 12.11%. Cephalosporins 69.89% were the most commonly prescribed antibiotic class. The number of antibiotics consumed was 11.08 DDD/100 patient days. Cefotaxime, with a DDD/100 patient days value of 2.95, was the most frequently prescribed antibiotic (47.72%). Evaluation of antibiotics uses based on WHO AWaRe 2021 showed that 31.6% and 68.4% of prescribed antibiotics were in the Access category and watch category, respectively.

**Conclusion:**

antibiotic use was high in the research setting. Over half of the antibiotic use was in the “Watch” group, according to the usage control criteria. Ceftazidime, cefixime, cefotaxime, and ceftriaxone had the highest levels of antibiotic consumption.

## Introduction

Amid the increasing levels of antimicrobial resistance, it is estimated that the UK would cause up to 110 million deaths per year by 2050, representing a 2%-3.5% drop in GDP, and a cost of up to $1100 trillion USD [1]. In Africa, it is estimated to cause 4.1 million deaths, while in Asia, this phenomenon would cause 4.7 million deaths, a 7% reduction in GDP, and a cost of up to $100 trillion USD [2]. The consumption of antibiotics increases in line with the changes in GDP growth. Worldwide, antibiotic use is estimated to more than double by 2030, compared to the figures in 2015 [3]. Irrational antibiotic use can result in antibiotic resistance, which, in turn, can lead to increased morbidity and mortality, as well as high treatment costs [4]. Antibiotic resistance is a crucial problem worsened by irrational antimicrobial consumption [5]. This phenomenon is more common in children because they are a population that often receives antibiotics. Approximately 60% of hospitalized pediatric patients received at least one type of antibiotic during their hospitalization, whereas approximately 20% of outpatient pediatric patients received antibiotics at inappropriate daily doses and for longer durations. Penicillins, cephalosporins, macrolides, and quinolones were the most widely prescribed antibiotics for pediatric patients in Europe during 1997 and 2003 [6,7]. Thus, to reduce the incidence of antibiotic resistance, antibiotic use in hospitals should be evaluated, and this can be reviewed objectively using the Anatomical Therapeutic Chemicals (ATC)/DDD method and qualitatively using the Gyssen criteria [8].

Gentamicin and ampicillin are two of the commonly prescribed antibiotics in pediatric patients, at the Bengkulu Indonesia Hospital. According to a quantitative analysis using the ACT/DDD index, the use of antibiotics for pediatric patients is higher than the levels recommended by the World Health Organization (WHO) [9]. By the way study of antibiotic consumption in two hospitals, namely, Orotta National Referral and Teaching Hospital and Hazhaz Zonal Referral Hospital, benzylpenicillin was the most commonly prescribed during the study period from 2014 to 2018. Furthermore, ceftriaxone and ciprofloxacin were among the antibiotics most commonly used in both hospitals [10]. The WHO proposed a categorization of antibiotics in March 2017 that includes access, watch, and reservations to maximize the rational use of antibiotics and enhance monitoring [11,12]. This categorization provides details on 180 antibiotics categorized as Access, Watch, and Reserve, as well as pharmacology, anatomical classification codes, and position on the WHO list of essential medicines (EML) [13].

At least one intravenous antibiotic was delivered to children in the outpatient and emergency departments of 16 tertiary care hospitals in China 2018. The most frequently administered antibiotic was azithromycin, while the most commonly prescribed antibiotic class consisted of third-generation cephalosporins. Furthermore, in Chinese pediatric outpatients, overuse and misuse of WHO Watch group antibiotics for respiratory tract infections and viral infectious illnesses are prevalent. Thus, the WHO AWaRe metrics should be used to improve pediatric antimicrobial stewardship [14]. The WHO expert committee on the selection and use of essential medicines established the AWaRe antibiotic classification in 2017 as a tool to aid antibiotic stewardship initiatives at the local, national, and global levels. Under this system, antibiotics are divided into three categories, namely, access, watch, and reserve, based on the influence of various antibiotics and antibiotic classes on antimicrobial resistance. The WHO´s 13^th^ general program of work (2019-2023) sets a country-level goal in which access group antibiotics should account for at least 60% of overall antibiotic use [15].

The access group of antibiotics are first- and second-line treatments for 21 common or severe clinical diseases. This comprises a core collection of antibiotics that should always be available in all areas at the appropriate quality, dosage, duration, formulation, and price. In comparison, the watch group of antibiotics has a higher risk of toxicity resistance than those in the access group. The watch group´s antibiotics contribute to the development of stewardship tools at the local, national, and global levels. When all other options fail, antibiotics in the reserve category are used as a last resort for specific individuals and clinical situations. Prioritizing this population as a primary focus of high-intensity national and international stewardship initiatives can help maintain their efficacy [12]. However, to date, only a few papers have been published in Indonesia related to the evaluation of antibiotic use with defined daily doses methods and WHO AWaRe classification. In this report, we presented 2020 data on antibiotic consumption at two referral hospitals in Central Java Province. The goal of this work was to evaluate antibiotic use in pediatric inpatients in Indonesia using the DDD method and the WHO AWaRe 2021.

## Methods

**Study design**: this is an observational study that uses a quantitative approach to calculate the quantity of antibiotic use in pediatric patients. This study used a cross-sectional design with retrospective data collection on pediatric inpatient medical records from January-December 2020.

**Study setting and population:** two hospitals in Central Java, Indonesia conduct an evaluation based on WHO AWaRe 2021. This study population was pediatric patient medical records from 2020. The sampling technique was purposive sampling. All patients who met the inclusion criteria and were received within the study duration were included in the study. The study´s inclusion criteria were as follows: hospitalized pediatric patients, diagnosed with an infection, aged 0-12 years, and readable. The exclusion criteria were applied to patients who left before being confirmed as fully cured or who died during treatment. Data is presented in tables and figures.

**Variables:** variable included social demographics (gender, age, duration of hospitalization), diagnosis and antibiotics (item of antibiotics, dosage and directions for use, duration of therapy antibiotics).

**Data resource and measurement:** observations conducted over six months collecting retrospective prescribing data antibiotics in pediatric inpatients in two hospitals in Central Java, Indonesia in January-June 2021.

**Data collection tool:** antimicrobial stewardship program data collection form.

**Data collection:** data were collected using a standard form of antimicrobial stewardship: patient personal information (sheet demographic variables such as sex, age, weight) date of hospital admission, hospital admission condition, ward, hospital discharge date, hospital discharge condition, diagnosis out, vital signs (HR, RR, temp). Important physical discovery, action, supporting investigation, laboratory, microbiological examination (culture), antibiotics and dosing regimen.

**Sample size:** pediatric patients hospitalized from January to December 2020 amounted to 4007 patients while fulfilling the inclusion criteria amounted to 505 medical records.

**Data analysis:** data obtained were assessed the use of antibiotics in a quantity which is calculated using the ATC/DDD index. Antibiotic data can be organized in a tabular format with the following fields: drug name, active substance name, drug class, ATC code, dosage form, dosage strength (in mg), and DDD/100 patient days. In 2020, the WHO collaborating center established guidelines for ATC/DDD classification. The first step in the analysis of the number of antibiotic prescriptions (ATC/DDD) is searching for the generic name and dosage strength of trademarked drugs based on Indonesian MIMS, followed by providing the code based on the ATC index with DDD 2019 and the DDD value based on the WHO standard. To calculate DDD/100 patient days, we collect data on antibiotic use in hospitalized pediatric patients and length of stay for inpatient care (total length of stay (LOS) for all patients in 2020). Then, we calculate the number of antibiotic doses (in grams) prescribed during therapy. The rational use of antibiotics was assessed using the WHO AWaRe 2021. This study is the first in the province, of Bengkulu in two hospitals in central Java, Indonesia related to the evaluation of the use of antibiotics. Therefore, follow-up research in the hospital sector was required to look at current prescribing procedures and then assess how well healthcare professionals perform when it comes to the rational use of medications (RUM). Therefore, a follow-up study in the government hospital sector was needed to investigate the current prescribing practices and subsequently evaluate the performance of healthcare providers in the rational use of medicines (RUM).

**Ethical consideration:** this study was approved by the Research Ethics Commission of the Faculty of Medicine, Gadjah Mada University, Yogyakarta (KE/FK/0960/EC/2020).

## Results

The results of this analysis covered 505 medical records that met the eligibility criteria.

### Social demographic analysis

**Data of hospitalized pediatric:** as shown in Table 1. Between January and December 2020, 50.89% of the 505 medical records of hospitalized pediatrics patients were male, and 49.11% were female. Grouping the data by age revealed that the age group of 0 to 28 days had a high infection rate at 31.61%, while the age group of >6 to <12 years had the lowest at 18.49%. In this research, the majority of patients (253, 50.30%) were treated for 1-4 days, while there were five (70.99%) with LOS data of at least 15-19 days.

**Table 1:** social demographic data of hospitalized pediatric patients

Characteristics	Frequency n(%)
**Gender**	
Male	256 (50.69)
Female	249 (49.31)
**Age**	
0-28 days	159 (31.49)
>1 month to ≤2 years	138 (27.33)
>2 years to ≤6 years	115 (22.77)
>6 years to ≤12 years	93 (18.42)
**Duration of hospitalization**	
1-4 days	253 (50.10)
5-9 days	222 (43.96)
10-14 days	17 (3.76)
15-19 days	5 (0.99)
>20 days	6 (1.19)

Characteristics pediatrics inpatients between January and December 2020

**Common diseases among pediatric inpatients:** the characteristics of the diagnosis of the disease were adjusted according to the international classification of diseases, tenth revision (ICD-10) version implemented by the WHO in 2010. After the adjustment, the results showed that the more common diseases among pediatric patients were as follows: unspecified fever (113), gastroenteritis and colitis (63), febrile convulsion (42), acute bronchiolitis (33), low birth weight (27), typhoid fever (23), birth asphyxia (22), acute respiratory infection (20) as shown in Table 2.

**Table 2:** common diseases among pediatric inpatients

Diagnosis	Frequency
Acute upper respiratory infections (J00-J06)	110
Gastroenteritis and colitis (A09.9)	65
Pneumonia (J18.9)	42
Bronchiolitis (J21.9)	33
Simple febrile convulsions (R56.00)	30
Typhoid fever (A01.00)	26
Urinary tract infection (N39.0)	22
Asphyxia (R09.01)	21
Dengue fever (A90)	14
Peritonitis (K65)	14
Diagnosis of pediatric patients based on ICD.10	

### Descriptive analysis

**Figure 1:**
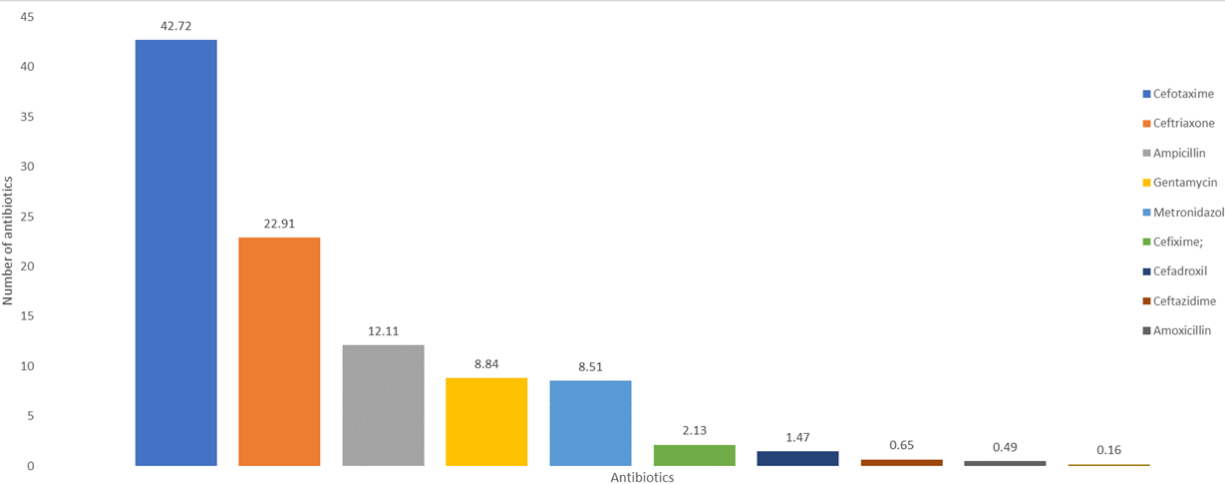
the frequency of antibiotic use among pediatric inpatients

**The frequency of antibiotic use among pediatric inpatients:** in Figure 1, cefotaxime accounted for 42.72% of the most commonly prescribed antibiotics in our pediatric hospital, followed by ceftriaxone (22.91%) and ampicillin (12.11%).

**The overall use of antibiotics in hospitalized pediatric patients** in Table 3 the results of quantitative analysis based on the LOS number of 3636 days revealed that ceftriaxone and ceftazidime had the highest and lowest values at 4.42 and 0.01, respectively. Most of the antibiotics prescribed to pediatric inpatients, including those under the watch criteria (68.41%) can be seen in Table 4, Table 5.

**Table 3:** values of DDD/100 patient days for each antibiotic and class, and the ATC code and WHO DDD standard

Antibiotics	Kode ATC	DDD (WHO)	DDD/100 patient days
Ampicillin	J01CA01	6	1.50
Amoxicillin	J01CA04	3	0.04
Ampicillin- sulbactam	J01CR01	6	0.02
Ceftazidime	J01DD02	4	0.01
Cefadroxil	J01DB05	2	0.03
Cefixime	J01DD08	0.4	0.41
Cefotaxime	J01DD01	4	2.95
Ceftriaxone	J01DD04	2	4.42
Gentamycin	JO10GB03	0.24	0.65
Metronidazole	P01AB01	2	1.07

ATC/DDD Data Based on WHO Collaborating Center 2020; ATC: anatomical therapeutic chemicals; DDD: defined daily doses

**Table 4:** classification of antibiotics according to the WHO’s AWaRe classification

Antibiotics	Class	AWaRe category	Frequency n (%)
Cefotaxime	Third-generation-cephalosporins	Watch	261 (42.72%)
Ceftriaxone	Third-generation-cephalosporins	Watch	140 (22.91%)
Ampicillin	Penicillins	Access	74 (12.11%)
Gentamycin	Aminoglycosides	Access	54 (8.84%)
Metronidazole	Imidazole	Access	52 (8.51%)
Cefixime	Third-generation-cephalosporins	Watch	13 (2.13%)
Cefadroxil	First-generation-cephalosporins	Access	9 (1.47%)
Ceftazidime	Third-generation-cephalosporins	Watch	4 (0.65%)
Amoxicillin	Penicillins	Access	3 (0.49%)
Ampicillin sulbactam	Beta-lactam/beta-lactamase-inhibitor	Access	1 (0.16)

Classification of antibiotics according to the WHO’s AWaRe Classification 2021; WHO: World Health Organization

**Table 5:** antibiotics presented according to WHO’s AWaRe classification

Categories	Frequency
Access	193 (31.59%)
Watch	418 (68.41%)

Antibiotics presented according to WHO’s AWaRe classification 2021; WHO: World Health Organisation

## Discussion

Hospitalized pediatric patients during the 2020 period involved 49 main diagnoses. The top three most frequently encountered diseases were acute upper respiratory, gastroenteritis and colitis and pneumonia comprising 21.78%, 12.87%, and 8.32%, respectively. These results are similar to a study conducted at the University Teaching Hospital in Lusaka, Zambia, which reported that most of the pediatric patients presented with respiratory diseases followed by gastrointestinal conditions, such as diarrhea [16]. Other studies conducted in India [17] and Ethiopia [18] reported similar results. Most of the diagnoses reported in the current study are similar to those reported in other studies, which can be attributed to the fact that respiratory tract infections are more infectious than other diseases, especially in poorly ventilated areas or dusty environments [19]. Furthermore, as young children begin crawling and teething, they are more likely to eat various dirty objects, thus increasing the risk of gastrointestinal tract infection [20]. Our results revealed that the most often prescribed antibiotics were cephalosporins and penicillin. Pradeepkumar *et al*. [21], Iftikhar *et al*. [22], and Mathew *et al*. [23] reported similar results indicating that these two are the most frequently prescribed antibiotics for infectious disorders, owing to their broad spectrum of activity, clinical effectiveness, and tolerance across all age groups [24]. Moreover, cephalosporins were among the most commonly used antibiotics at our centre, thus matching findings from previous research concluding that cephalosporins in general (and ceftriaxone in particular) were among the most commonly used antibiotics [24-27].

The WHO recommends the quantitative evaluation and measurement of antibiotic use, which may be conducted using ATC and DDD, respectively. DDD is only applied to medications that already have an ATC code. The ATC/DDD method has the advantage of simplicity compared to other institutions at the national and global levels. In particular, DDD is a method for transforming and standardizing drug quantity data into an estimate of drug usage in a healthcare setting [28]. According to the results of the DDD value calculation in the table above, ceftriaxone and ceftazidime had the highest and lowest values at 4.42 and 0.01 DDD/100 patient days, respectively. Based on the DDD/100 patient days calculation results, some antibiotics, including ceftriaxone and gentamicin, have higher values than the standard value set by the WHO. When the quantity of antibiotic use stated in the DDD value is higher and does not meet the standard WHO value, this indicates that the prescription and use of antibiotics in patients may not be selective. Unfortunately, many prescriptions and uses of antibiotics that are not properly indicated will affect the rationale for the use of such antibiotics. Some high values of DDD/100 patient days for several types of antibiotics, which exceed the WHO standard value, indicate that there may still be irrational antibiotic use in hospitalized pediatric patients in Indonesia in 2020. Apart from the possibility of using antibiotics for inappropriate indications, due to the excess dosage, a high DDD value of several types of antibiotics exceeding the WHO standard is also an early indication of the possibility of giving antibiotics at excessive dosages. Furthermore, the DDD/100 value calculation results showed that gentamicin had a total value of 0.65, while ceftriaxone had a total DDD value of 4.42, which exceeded the WHO standard values of 0.24 and 2, respectively.

Gentamicin is typically used in conjunction with other antibiotics. In pediatric treatment, once-daily gentamicin medication is becoming more prevalent, although little is known regarding its pharmacokinetics in critical illness. Given that gentamicin kills in a concentration-dependent manner, peak serum concentrations at least eight times greater than the target organism´s minimum inhibitory concentration have been proposed [29]. This study reported the use of gentamicin in combination with ampicillin or cefotaxime. The European studies on the antibiotic consumption monitoring project (ESAC) reports that antimicrobial combinations have been used in 65% of systemic infection treatments, the most frequent of which is the combination of penicillin with aminoglycosides, especially gentamicin (21%) [30]. Ceftriaxone is widely used in pediatric patients of all ages [31]. For example, a study in Turkey reported ceftriaxone and ampicillin sulbactam were ranked first and second among the most commonly used antimicrobials in a 50-bed pediatric ward of a tertiary research hospital [32]. In the treatment of severe sepsis, ceftriaxone is a useful broad-spectrum antibiotic. Indeed, it is one of several antibiotics with a broad spectrum of action. Even though it appears to be reversible, the increased incidence of biliary pseudolithiasis and cholelithiasis is a worrying development. Ceftriaxone toxicity in children is most commonly caused by GI ADRs, with the most prevalent ADRs in the GI tract being diarrhoea, nausea, and vomiting. The most significant ADRs and the main causes of ceftriaxone discontinuation in children are biliary pseudolithiasis and immunological hemolytic anaemia. Almost all cases of biliary pseudolithiasis may be reversed. Meanwhile, children with sickle cell disease are more prone to develop immune hemolytic anaemia, which can lead to death. As a result, ceftriaxone should be taken with caution among children with sickle cell disease [33].

In our study, we prescribed six antibiotics from the Access group and four medicines from the Watch category. The Access group includes at least 60% of the antibiotics consumed, according to the WHO´s AWaRe categorization [34]. Furthermore, antibiotics from the Access category were prescribed 31.59% of the time based on our results. Meanwhile, antibiotics from the Watch group accounted for 68.41% of all antibiotics given, suggesting that those given under the Watch category are being overused. A study of pediatric antibiotic prescriptions in China discovered a similar trend of misuse. According to a pediatric survey, different nations consume varying quantities of AWaRe antibiotics [14]. In Slovenia, 61.2% of children in the Access group received antibiotics compared to 7.8% in China. The highest prevalence of antibiotic use among children (77.3%) is found in Iran, while the lowest rate is found in Finland (23.0%). In neonates, Singapore had the greatest usage of access group antibiotics, accounting for 100% of all prescriptions, whereas China had the lowest use at 24.2% [35]. Furthermore, according to the WHO Watch group, misuse of broad-spectrum antibiotics to treat respiratory infections and viral infections in Chinese people is a severe health problem. To monitor and manage antibiotic use, the WHO AWaRe metrics and indicators for antibiotic use should be used as antimicrobial stewardship tools and evaluation indicators [14].

**Limitation:** this research was conducted in a short time because the research schedule was determined by the hospital where the data was collected. Hospitals that are evaluated for antibiotic prescribing do not yet use a computerized drug registration system so there may be missing data and the method utilized is retrospective, although this approach has a flaw in that medical records are not written completely.

## Conclusion

This study found a high level of antibiotic users in the selected research setting. Over half of the antibiotic use were in the Watch group according to the usage control criteria. Finally, ceftazidime, cefixime, cefotaxime, and ceftriaxone had the highest levels of antibiotic consumption.

### What is known about this topic

- *Antibiotic resistance is a crucial problem worsened by irrational antimicrobial consumption; this phenomenon is more common in children because they are a population that often receives antibiotics;*
- *Approximately 60% of hospitalized pediatric patients received at least one type of antibiotic during their hospitalization, whereas approximately 20% of outpatient pediatric patients received antibiotics at inappropriate daily doses and for longer durations*.

### What this study adds

- *Cefotaxime, ceftriaxone and ampicillin are the most commonly prescribed antibiotics in pediatric inpatients;*
- *Most of the antibiotics prescribed to pediatrics inpatients, including those under the watch criteria;*
- *Antibiotic use in children is grouped into AWaRe categories: access, watch, and reserve*.
